# Contraceptive discontinuation: frequency and associated factors among undergraduate women in Brazil

**DOI:** 10.1186/s12978-019-0783-9

**Published:** 2019-08-29

**Authors:** Christiane Borges do Nascimento Chofakian, Caroline Moreau, Ana Luiza Vilela Borges, Osmara Alves dos Santos

**Affiliations:** 1 Direct-Entry Midwifery Program, School of Arts, Sciences and Humanities of the University of São Paulo, Av. Arlindo Bettio, 1000 – Ermelino Matarazzo, São Paulo, São Paulo 03828-000 Brazil; 2 Department of Population, Family and Reproductive Health, Johns Hopkins Bloomberg School of Public Health, Baltimore, MD USA; 3 Gender, sexual and reproductive health, CESP Centre for research in Epidemiology and Population Health, U1018, Inserm, F-94807 Le Kremlin-Bicêtre, France; 4 Department of Public Health Nursing, University of São Paulo School of Nursing, São Paulo, Brazil

**Keywords:** Contraception, Contraceptive discontinuation, College students, Sexual and reproductive health, Brazil, Contracepção; Descontinuidade Contraceptiva; Universitárias; Saúde Sexual e Reprodutiva; Brasil.

## Abstract

**Background:**

In Brazil, high contraceptive prevalence rates coexist with high rates of unintended pregnancies. Contraceptive discontinuation may explain this context, but few studies have focused on highly educated young women in countries with low unmet need for modern contraception. This paper explores frequency and associated factors of contraceptive discontinuation among undergraduate students in Brazil within 12-months.

**Methods:**

This retrospective cohort study was conducted among a probability sample of 1679 undergraduates of São Paulo University. Data were collected online using a contraceptive calendar. We examined factors related to monthly discontinuation of oral pills and male condoms using Generalized Estimating Equation models.

**Results:**

Altogether, 19% of oral pill users and 48% of male condom users discontinued their method for method-related reasons within 12-months, and 18% of oral pill users and 15% of male condom users abandoned/or switched to less effective methods. Women in casual relationships were at increased odds of oral pill (OR = 1.4 [1.1–1.8]) and male condom discontinuation (OR = 1.3 [1.0–1.7]), and at increased odds of switching from oral pill to less effective or no method (OR = 1.4 [1.1–1.7]). Other associated factors were method specific. Women from lower socioeconomic status or who had multiple lifetime partners were more likely to discontinue or abandon the oral pill, while more sexually experienced women were less likely to discontinue the male condom.

**Conclusion:**

Frequent method discontinuation in Brazil calls for greater attention to the difficulties women face when using short acting methods. Discontinuation was associated with type of partner and sexual experience highlighting the changing contraceptive needs of women at the early stages of their professional careers.

## Resumo

**Introdução:**

No Brasil, as altas taxas de prevalência de métodos contraceptivos coexistem com as altas taxas de gravidezes não desejadas. A descontinuidade dos métodos contraceptivos pode explicar esse contexto; entretanto, poucos estudos focam mulheres jovens com alto nível educacional, em países que apresentam baixo número de mulheres com necessidade insatisfeita por métodos contraceptivos modernos. Desse modo, o presente estudo explora a frequência e os fatores associados das descontinuidades contraceptivas entre estudantes universitárias no Brasil, em um período de 12 meses.

**Métodos:**

Estudo de coorte retrospectivo realizado com uma amostra probabilística de 1679 graduandas da Universidade de São Paulo, Brasil. Os dados foram coletados online usando um calendário contraceptivo. Para examinar os fatores relacionados à descontinuidade mensal das pílulas orais e dos preservativos masculinos foram utilizados os modelos de Equações de Estimação Generalizadas.

**Resultados:**

No total, 19% das usuárias da pílula oral e 48% das usuárias do preservativo masculino descontinuaram o método por razões relacionadas ao método em um período de 12 meses, e 18% das usuárias da pílula oral e 15% das usuárias do preservativo masculino abandonaram ou mudaram para um método menos eficaz. As mulheres que tinham relacionamentos casuais tiveram mais chance de descontinuar a pílula oral (OR = 1,4 [1, 8]) e o preservativo masculino (OR = 1,3 [1,0–1,7]), e tiveram mais chance de mudar a pílula oral para um método menos eficaz ou para nenhum método (OR = 1,4 [1, 7]). Outros determinantes foram específicos do tipo do método contraceptivo. As mulheres de nível socioeconômico mais baixo ou que tiveram múltiplos parceiros sexuais durante a vida tiveram uma maior probabilidade de descontinuar ou abandonar a pílula oral; todavia, as mulheres que tinham mais experiências sexuais foram menos propensas a descontinuar o preservativo masculino.

**Conclusão:**

A frequente descontinuidade contraceptiva no Brasil chama atenção para as dificuldades que as mulheres enfrentam quando usam métodos de curta duração. A descontinuidade contraceptiva foi associada ao tipo de parceiro e à experiência sexual, destacando a necessidade de mudanças contraceptivas entre mulheres que estão nos estágios iniciais de suas carreiras profissionais.

## Plain English summary

Contraceptive discontinuation is defined as switching a method of contraception or stopping a method altogether. In this study, a probabilistic sample of undergraduate women from University of São Paulo/Brazil answered a contraceptive calendar via an online survey. Respondents were asked about the main contraception that they used in the past 12-months. For each month in the calendar, the student provided information about the reasons for discontinuation, occurrence of pregnancy and their relational status with a partner. Of the 1679 respondents: 19% of oral pill users and 48% of condom users discontinued their method for method-related reasons within 12-months, and 18% of pill users and 15% of condom users abandoned contraceptive use or switched to a less effective method (e.g., from oral contraceptives to male condom or from male condom to withdrawal). Changes in patterns of contraceptive use differed by characteristics of the respondents. Women of lower socioeconomic status and those who had had more than one sexual partner in their lifetime were more likely to stop using the pill, while more sexually experienced women (those for whom more than 1 year had passed since first sexual intercourse) were less likely to discontinue condom use. The major finding of the research was that the type of relationship, socioeconomic status, sexual experience, sexual partners, and type of contraceptive were strongly associated with contraceptive discontinuation among undergraduate students in Brazil.

## Background

Brazil has recently undergone a rapid fertility transition with total fertility rates dropping below replacement, from 6.3 in 1986 to 1.8 in 2006, and parallel increase in modern contraceptive prevalence rates from 57% in 1986 to 77% in 2006 [1, 2]. Despite dramatic advances in contraceptive coverage and low unmet need for modern contraception (6%), 55.4% of pregnancies were reported as unintended in Brazil [3], a situation that is comparable to other countries with high contraceptive coverage [4–6].

In countries with moderate to high contraceptive prevalence, the majority of unintended pregnancies occur as a result of contraceptive discontinuation (defined as switching a method of contraception, stopping a method altogether, or failures due to inconsistent use) [7–9]. Considering all methods, 27.8% of all methods are discontinued for method-related reasons within 12-months in Brazil; more specifically, 21.6% of women switching their method and 10.3% abandon the method within the first year of use [10]. In this scenario, it is important to highlighted that although switching can heighten the risk of an unintended pregnancy, since failures are higher at the start of use of a method when women are still familiarizing themselves with the regimen, the risk of an unintended pregnancy are higher when a woman abandon the method or switch to less effective method [11].

Contraceptive discontinuation is associated with many factors, including external factors (such as quality of family planning services, guidelines, among others), but also individual and partner factors (such as demographic characteristics, partner dissatisfaction, fertility motivations, among others) [12, 13]. A study carried out with Brazilian women observed that method switching was more common among married users than their unmarried counterparts, and abandonment was positively associated with the parity, and inversely associated with educational level [10].

Contraceptive discontinuation is an important contributing factor to unintended pregnant and induced abortion. In the Brazilian context, where induced abortion is highly restricted, and thus often practiced in clandestine and unsafe contexts, this means that contraceptive discontinuation also contributes to maternal mortality and morbidity. Understanding contraceptive discontinuation is significant because it has negatively and positively reproductive consequences for women throughout their life course, especially among young women who the contraceptive trajectories are dynamic [14]. In addition, from the life course perspective, comprehending contraceptive and reproductive behaviors among young people are important, because family (and union) formation has experienced a major transformation over the last decade with delays in marriage [15, 16]. It means that a growing population of youth exposed to a potential risk of unintended pregnancy.

The latest data on discontinuation which mention youth in Brazil, dates back to the 1996 Demographic and Health Survey (DHS) [1] with little insights on how overall practices and user dynamics have evolved ever since. In Brazil, the method mix of contraceptives (especially long-acting reversible contraceptive methods (LARCs)) is limited. Consequently, among young women in Brazil, pills and male condoms dominate the contraceptive landscape, but they are usually associated with high discontinuation rates and moderate effectiveness [1, 8, 11, 17, 18]. Undergraduate students are especially motivated to delay childbearing until reaching professional stability [19, 20]. While they have lower pregnancy rates compared to less educated women [1, 6, 21], they are also more likely to report their pregnancies as unintended (60%), most of which are due to contraceptive discontinuation [22, 23]. In addition, highly educated Brazilian young women only represent 12.6% of women of reproductive age and therefore [16], their specific sexual and reproductive behaviors and needs are not well captured in a population based study including all women of reproductive age, such as the DHS. Therefore, the purpose of this study was to explore the frequency and the factors associated to contraceptive discontinuation for method-related reasons, and abandonment or switching to less effective method among undergraduate women.

## Methods

We conducted a 12-months retrospective cohort study among a sample of undergraduate women from a public university in Brazil. São Paulo University is the largest institution of higher education and research in the country and best ranked in Latin America. The study obtained approval from the Human Research Ethics Committee of University of São Paulo School of Nursing.

Women aged 18–24 enrolled in regular undergraduate courses were eligible. They were selected by simple random sampling without replacement, based on the list of all the email addresses of students provided by the university (*n* = 18,193 emails from different female students). The sample included 1026 women, however considering the low response rate (10%) in the pre-test (*n* = 50), the sample was tripled. Thus, an online questionnaire was sent to 3078 women selected from the email list. A total of 2182 (71%) responded to the email, from which 50 refused to participate; and 453 were deemed non-eligible due to age (< 18 (*n* = 2)), sexual activity (*n* = 358 had never had sexual intercourse), and contraceptive use in the previous 12-months (*n* = 93 had never used contraception in the last 12-months). Our final population included 1679 female students aged 18–24 who reported they had ever had sexual intercourse and who had used contraception in the past 12-months.

Data were collected online through *Google Form*, using a self-administered questionnaire; which took, on average, 5–7 min to complete. The sociodemographic characteristics included: age (18–19/20–24); race/ethnicity (white/black/others); religious affiliation (no religion/catholic/Kardecist Spiritualism/Evangelical/others); relationship status (steady/casual); socioeconomic status (A/B: high level of income, and C–D/E: middle or low level of income) [24]; field of study (Human/Health/Exact Sciences); type of student enrollment (full-time/part-time); and campus site (São Paulo/out of São Paulo). Sexual reproductive information included: time since first sexual intercourse (≤1 year/2–3 years/≥4 years); number of sexual partners (1 partner/2–3 partners/≥4 partners); and previous pregnancy (no/yes). Women also completed a contraceptive calendar on a month-by-month basis covering the previous 12-months. The calendar was derived from the DHS survey [9], and collected monthly information on partnership status, contraceptive method used, and reasons for discontinuation if women reported different methods two consecutive months. Users of coital dependent methods were asked to indicate if they still considered themselves as using their method even in months when they had no sexual activity in order not to conflate coital method discontinuation with the absence of sexually activity [25].

Therefore, in this context, we defined two measures of discontinuation, which represent different levels of risk relative to unintended pregnancy. The first measure, “discontinuation for method-related reasons,” classifies all women who discontinue their method but are still in need of contraception (with a partner, not pregnant, not trying to conceive, not sterile) as having discontinued for method-related reasons. The second, “abandoned or switched to less effective method”, includes women who switch to a less effective method within a month or stop their method altogether while still in need of contraception [26]. Switching to a less effective method was defined as either switching from oral contraceptives to barrier methods, spermicide, fertility awareness, or withdrawal; or switching from male condom to withdrawal, fertility-awareness, or spermicide [17]. We combined abandonment and decreased efficacy in a single category due to the very small number of women who reported abandoning their method. While pregnancy risk is higher for non-users as compared to users of other less effective methods, we considered that the drop in contraceptive efficacy was an important indicator of contraceptive use dynamics [17, 27, 28].

### Statistical analysis

We first described patterns of contraceptive use according to method type (pill, male condom) at the start of the contraceptive calendar (12-months prior to the survey, which we consider to be baseline). We focused on pill and condoms because together they represent 95% of the method mix in this population; thus, we did not consider other methods, such as withdrawal/fertility-awareness/spermicide/diaphragms (*n* = 91 women). We then turned to method specific monthly-level analysis of pill and condom discontinuation, examined across 12,378 months of pill use and 3581 months of male condom use. We explored bivariate associations between women’s sociodemographic characteristics, their sexual and reproductive health characteristics and their monthly probability of discontinuing the pill or the condom, according to our two indicators of discontinuation. In this analysis, partnership type was a monthly time varying measure. We then conducted multivariate analysis using a Generalized Estimating Equation (GEE) to investigate the odds of monthly discontinuation of the pill and condom (using both discontinuation indicators), while accounting for intra-correlation among months belonging to the same woman. We used an exchangeable correlation structure with logit link transformation function. The selection of the most appropriate correlation structure was based on the Quasi-likelihood under the Independence Model Criterion (QIC). Adjusted odds ratios and 95% confidence intervals were estimated in both models.

## Results

At baseline, women were on average 21 years old, 80.6% were white and 65.6% were in a stable relationship. The average age of sexual debut of these women was 17 years old, and 37 (2.2%) mentioned having been pregnant in the past, with 24 of these pregnancies ending in any abortion. Most women were using contraception at survey baseline (94.8%) with 67.2% using the pill, 22.5% using condoms, 5.1% using less effective methods (withdrawal/fertility-awareness/spermicide/diaphragms), and 5.2% reporting no use (data not shown in Table 1).

**Table 1:** Sociodemographic and sexual and reproductive health characteristics by type of contraceptive method used at baseline. São Paulo, Brazil – 2015

Variables	Total	Oral pill (*N* = 1190)	Condom (*N* = 398)	p*
	N	%	%	
Sociodemographic characteristics				
Age (years)				**< 0.001**
18–19	379	66.8	33.2	
20–24	1209	77.5	22.5	
Race/ethnicity				**0.018**
White (caucasian)	1280	76.4	23.6	
Black	217	67.7	32.3	
Other race/ethnicity**	91	71.4	28.6	
Religion				**0.025**
No religion	728	73.5	26.5	
Roman Catholic	501	77.1	22.9	
Kardecist Spiritualism Doctrine	196	81.1	18.9	
Evangelical	97	69.1	30.9	
Other religion***	66	65.2	34.8	
Type of relationship				**0.001**
Steady	1044	77.5	22.5	
Casual relationship or None	544	70.0	30.0	
Level of status				0.099
A/B	1259	75.9	24.1	
C – D/E	329	71.4	28.6	
Educational background				
Field of study				0.089
Human Sciences	643	72.3	27.7	
Health Sciences	588	75.7	24.3	
Exact Sciences	357	78.4	21.6	
Period of study				0.105
Full-time	814	76.7	23.3	
Part-time (morning, afternoon and evening)	774	73.1	26.9	
Campus				**< 0.001**
São Paulo	1039	71.8	28.2	
Out of São Paulo	549	19.1	80.9	
Sexual, reproductive and contraceptive behavior				
Time since first sexual intercourse				**< 0.001**
≤ 1 years	312	66.0	34.0	
2–3 years	561	74.0	26.0	
≥ 4 years	715	79.6	20.4	
Number of sexual partners in lifetime				**0.005**
1 partner	576	79.2	20.8	
2–3 partners	455	70.3	29.7	
≥ 4 partners	557	74.3	25.7	
Previous pregnancy				0.321
No	1554	75.1	24.9	
Yes	34	67.7	32.3	
Total	**1588**	**74.9**	**25.1**	

*Pearson’s chi-square test

**Race/ ethnicity include: asian origin and indigenous people

***Other religions include: Afro-Brazilian, Buddhism, Jewish, Muslim, Mormon, and Islam

**Fig. 1:**
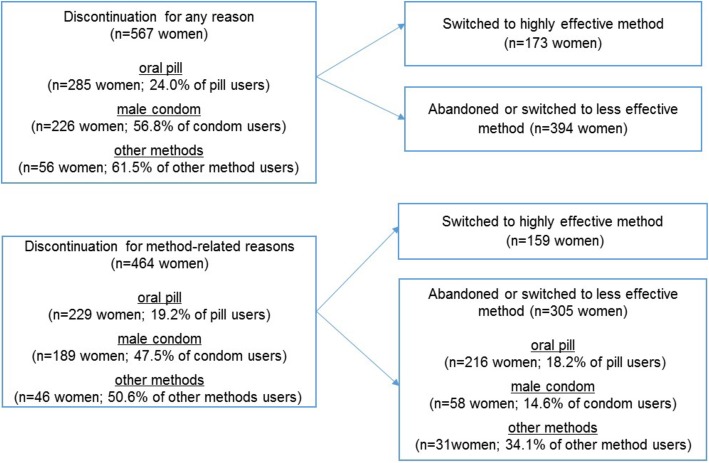
Number of women discontinuing their method in the last 12-months, by measure of discontinuation and type of method used at the start of the contraceptive calendar (baseline). São Paulo, 2015

Among the 1679 women in the sample, 27.63% (*n* = 464) discontinued their method for method-related reasons and 18.2% (*n* = 305) abandoned or switched to a less effective method (Fig. 1). Pills more likely to be used by students who were older, white, had more years of sexual experience, who only had one lifetime partner, and who were in steady relationships. Conversely, students who are younger, black, in casual relationships, have more than one lifetime sexual partner, and more recently began having sex were more likely to rely on condoms at baseline (Table 1).

Among a total of 12,378 months of pill use, 2.7% of monthly-events represented discontinuation for method-related reasons. In addition, 3.3% of monthly-events represented switching from the pill to a less effective method or to no method. Among a total of 3581 months of condom use, 9.5% of monthly-events represented discontinuation for method-related reasons. In addition, 5.2% of monthly-events represented switching from the condom to a less effective method or to no method. Most women discontinued the pill because of side effects (66%), while condom users discontinued to switch to more effective methods (62%) or because their partners disliked the method (18%) (data not shown in Table).

Bivariate analyses of factors related to monthly discontinuation are reported in Table 2. Results indicate factors related to method discontinuation and abandonment/or switch to less effective contraception were mostly similar for both methods. The exception was time since first sex, which it was significantly related to discontinuation but not to method abandonment. Women who had been pregnant before were more likely to discontinue condoms but not to abandon condoms for less effective or no methods. In addition, condom users who were younger were more likely to abandon contraceptive use or switch to a less effective contraception (e.g., withdrawal, fertility-awareness, or spermicide). Results from the GEE multivariate models presented in Table 3, show that partnership type was the strongest factor related to monthly discontinuation. Pill and condom users in casual relationships were at increased odds of monthly discontinuation. Furthermore, a prior pregnancy increased the odds of pill and condom discontinuation. Other determinants were method specific. For instance, pill users of lower socioeconomic status or who reported multiple lifetime partners (more than one sexual partner in lifetime) had higher odds to discontinue or abandon/switch to less effective method. In contrast, women decreased odds to discontinue the condom as they became more sexually experienced (as measured by years since first sexual intercourse).

**Table 2:** Proportion of all months of contraceptive discontinuation according to the type of discontinuation, by type of contraceptive method. São Paulo, Brazil – 2015

Variables	Oral Pill	Condom	Discontinuation for method-related reasons				Abandonment or switching to less effective method			
			Oral Pill		Condom		Oral Pill		Condom	
	N	N	%	p*	%	p*	%	p*	%	p*
Sociodemographic characteristics										
Age (years)				0.301		0.874		0.489		**0.032**
18–19	2664	1053	2.4		9.4		3.1		6.5	
20–24	9714	2528	2.7		9.6		3.4		4.7	
Race/ethnicity				0.137		0.165		0.501		0.661
White (caucasian)	10,186	2719	2.6		9.9		3.2		5.4	
Black	1515	660	3.3		7.6		3.8		4.5	
Other race/ethnicity**	677	202	1.9		10.4		3.4		4.9	
Religion				**0.029**		0.348		**0.029**		0.817
No religion	5554	1776	2.7		8.9		3.5		5.1	
Roman Catholic	102	968	2.2		9.9		2.8		5.0	
Kardecist Spiritualism Doctrine	55	358	2.7		10.3		3.7		5.3	
Evangelical	23	260	2.9		8.1		3.4		5.8	
Other religion***	180	219	4.7		12.8		5.4		6.8	
Type of relationship at the time of discontinuation				**< 0.001**		**0.054**		**< 0.001**		**0.052**
Steady	8733	2179	2.2		8.8		2.8		5.4	
Casual relationship	3645	1402	3.7		10.7		4.6		4.9	
Level of status				**0.020**		0.473		**0.032**		0.315
A/B	9946	2705	2.5		9.7		3.2		5.4	
C – D/E	2432	876	3.3		8.9		4.0		4.6	
Educational Background										
Field of study				**0.045**		0.510		0.234		0.563
Human Sciences	4822	1591	3.1		10.1		3.6		5.3	
Health Sciences	4662	1310	2.3		8.8		3.0		5.5	
Exact Sciences	2894	680	2.5		9.4		3.3		4.4	
Period of study				**0.002**		0.830		**0.037**		0.907
Full-time	6514	1700	2.2		9.4		3.0		5.2	
Part-time	5864	1881	3.1		9.6		3.7		5.3	
Campus				**0.007**		0.134		**0.043**		0.340
São Paulo	7761	2672	2.9		9.1		3.4		5.0	
Out of São Paulo	4617	909	2.1		10.8		3.2		5.8	
Time since first sexual intercourse				**0.046**		**0.050**		0.314		0.927
≤ 1 years	2099	685	2.1		12.0		2.8		5.4	
2–3 years	4372	1368	2.4		8.8		3.3		5.0	
≥ 4 years	5907	1528	3.0		9.1		3.5		5.3	
Number of sexual partners in lifetime				**< 0.001**		0.666		**< 0.001**		0.612
1 partner	4783	1011	1.6		9.2		2.0		4.6	
2–3 partners	3355	1079	2.7		10.2		3.5		5.6	
≥ 4 partners	4240	1491	3.8		9.3		4.7		5.4	
Previous pregnancy				**< 0.001**		**0.053**		**0.001**		0.320
No	365	3482	2.5		9.4		3.2		5.3	
Yes	18	99	7.7		15.1		7.2		3.0	
Total	**12,378**	**3581**	**2.7**		**9.5**		**3.3**		**5.2**	

*Pearson’s chi-square test

**Other race/ ethnicity include: asian origin and indigenous people

***Other religions include: Afro-Brazilian, Buddhism, Jewish, Muslim, Mormon, and Islam

**Table 3:** Correlates of contraceptive discontinuation according to the type of discontinuation, by type of contraceptive method. São Paulo, Brazil – 2015

Variables	Discontinuation for method-related reasons		Abandonment or switching to less effective method	
	Oral Pill	Condom	Oral Pill	Condom
	OR_adjust_ (CI)*	OR_adjust_ (CI)*	OR_adjust_ (CI)*	OR_adjust_ (CI)*
Sociodemographic characteristics				
Age (years)				
18–19	1.0	1.0	1.0	1.0
20–24	1.0 (0.8–1.4)	1.2 (0.9–1.7)	1.1 (0.8–1.4)	0.7 (0.5–0.9)**
Race/ethnicity				
White (caucasian)	1.0	1.0	1.0	1.0
Black	1.1 (0.8–1.6)	0.7 (0.5–1.1)	1.1 (0.8–1.5)	0.9 (0.6–1.3)
Other race/ethnicity****	0.7 (0.4–1.3)	1.0 (0.6–1.8)	1.0 (0.6–1.5)	0.9 (0.5–1.8)
Religion				
No religion	1.0	1.0	1.0	1.0
Roman Catholic	0.9 (0.7–1.3)	1.1 (0.8–1.6)	0.9 (0.7–1.2)	0.9 (0.7–1.3)
Kardecist Spiritualism Doctrine	1.1 (0.8–1.6)	1.1 (0.7–1.7)	1.2 (0.9–1.6)	1.1 (0.6–1.7)
Evangelical	1.3 (0.8–2.1)	1.0 (0.6–1.7)	1.2 (0.8–1.9)	1.3 (0.7–2.2)
Other religion*****	1.6 (0.9–2.7)	1.5 (0.9–2.6)	1.5 (0.9–2.4)	1.4 (0.8–2.5)
Type of relationship at the time of discontinuation				
Steady	1.0	1.0	1.0	1.0
Casual relationship	1.4 (1.1–1.8)**	1.3 (1.0–1.7)**	1.4 (1.1–1.7)**	0.8 (0.6–1.1)
Level of income				
A/B	1.0	1.0	1.0	1.0
C – D/E	1.4 (1.0–1.8)**	0.9 (0.7–1.3)	1.3 (1.0–1.7)**	0.9 (0.7–1.3)
Educational background				
Field of study				
Human Sciences	1.0	1.0	1.0	1.0
Health Sciences	0.9 (0.7–1.3)	0.8 (0.5–1.2)	1.0 (0.7–1.3)	1.1 (0.7–1.6)
Exact Sciences	1.1 (0.7–1.5)	0.8 (0.6–1.3)	1.0 (0.8–1.4)	0.8 (0.5–1.2)
Period of study				
Full-time	1.0	1.00	1.0	1.0
Part-time	1.2 (0.9–1.7)	0.9 (0.6–1.3)	1.2 (0.9–1.5)	1.0 (0.7–1.5)
Campus				
São Paulo	1.0	1.0	1.0	1.0
Out of São Paulo	0.8 (0.6–1.0)**	1.3 (0.9–1.7)	1.0 (0.8–1.2)	1.3 (0.9–1.9)
Sexual and reproductive behavior				
Time since first sexual intercourse				
≤ 1 year	1.0	1.0	1.0	1.0
2–3 years	0.9 (0.6–1.3)	0.5 (0.4–0.9)**	0.9 (0.7–1.3)	0.9 (0.6–1.4)
≥ 4 years	0.9 (0.6–1.3)	0.6 (0.4–0.9)**	0.7 (0.5–1.1)	1.1 (0.7–1.7)
Number of sexual partners in lifetime				
1 partner	1.0	1.0	1.0	1.0
2–3 partners	1.7 (1.2–2.4)**	1.1 (0.7–1.6)	1.8 (1.4–2.5)**	1.3 (0.9–2.0)
≥ 4 partners	1.2 (1.6–3.2)**	0.9 (0.6–1.3)	2.4 (1.8–3.3)**	1.3 (0.9–2.1)
Previous pregnancy				
No	1.0	1.0	1.0	1.0
Yes	2.7 (1.6–3.7)**	1.8 (0.9–2.6)**	2.1 (1.3–3.6)**	0.6 (0.2–1.8)

*OR: odds ratios estimated using Generalized Estimating Equations with exchangeable matrix and logit link function assumed. CI: Confidence Interval

**p ≤ 0.050

***Other race/ ethnicity include: asian origin and indigenous people

****Religions include: Roman Catholic, Kardecist Spiritualism Doctrine, Evangelical, Afro-Brazilian, Buddhism, Jewish, Muslim, Mormon, and Islam

## Discussion

This is the first study to provide estimates of frequency of discontinuation among undergraduate women in Brazil, using a retrospective calendar data. It is original in providing estimates of switching and abandonment of contraceptive method. We focus our analysis on monthly odds of method discontinuation among women who have used contraception within 12-months period and who remain at risk of unintended pregnancy in order to identify sociodemographic and sexual health characteristics that are related to method discontinuation, which it can contribute increased risk of unintended pregnancy.

Our data shows that discontinuation is a relatively frequent event among undergraduate women in Brazil: almost three out of 10 women had at least one episode of discontinuation for method-related reasons, while two out of 10 women abandoned their method or switched to less effective method. Although our sample is different from 1996 DHS [18] (the sampling strategy involved geographical strata and it was representative of the country as a whole), and therefore comparison is limited, we found similar patterns of discontinuation. Nowadays, more women use contraception than do in 1996; however, this may indicate that discontinuation has changed little over the years in Brazil, despite upgraded health services and improvement in some women’s health indicators [29].

These relatively high rates of discontinuation are likely related to the method mix that are shown to have higher discontinuation rates [26]. Lack of professional training and limited contraceptive options [30, 31] reflect the difficulty for women to maintain contraceptive use or to adopt a new method after stopping their previous method. In addition, theses factors also contribute to a skewed method mix favoring short acting user dependent methods, especially among youth in Brazil who are at the peak of their fertility [30]. However, we emphasize that the desire for a more effective method represents a positive outcome of condom discontinuation for method-related reasons.

As previously reported in studies conducted in the U.S. or in France [28, 32, 33], countries with moderate to high contraceptive prevalence such as in Brazil, condom users were more likely to discontinue their method than pill users, but the reverse was true in case of method abandonment/switching to a less effective method. These results indicate the need to consider several indicators of discontinuation in order to better capture transitions that put women at risk of pregnancy from transitions that potentially improve pregnancy prevention. Regardless of the method used, partnership was the most significant variable of method discontinuation and abandonment. Other studies have pointed to the strong connection between partnership and contraceptive use dynamics. Among couples, condoms are mainly used during casual relationships or in the beginning of a relationship to prevent against Sexually Transmitted Infections (STIs) and are replaced by more effective contraception in more stable relationships [25, 34]. As relationships become more stable, many couples switch to hormonal methods, and the main reason to switch is the “supposed mutual trust”. It denotes that they become less concerned about the prevention of the STIs, which it still remains an important public health problem in Brazil [35].

Our analysis also indicates that discontinuation is higher in casual relationships. These findings align with work conducted by Sassler and colleagues [36], which indicates that couples that believe they have a future together are more consistent users. Other research in the US, however, notes that women living with their sexual partner are more likely to have gaps in contraception than women who do not live with their sexual partner [37]. Study population and context may account for part of the differences observed between studies, but all studies draw attention to partner effects [12].

We also found that as the number of sexual partners increased, the odds of pill discontinuation increased. This result may relate to our prior discussion about the need for STI prevention with new partners, which may lead some women to switch from pills to condoms when they start a new relationship [34]. In contrast, condom users at the start of their sexual lives (less than 1 year of sexual experience) were more likely to discontinue, a finding that corroborates the results of a previous study in Honduras [38].

As expected, discontinuation of condoms was mainly related to criticism of the partner and wanting a more effective method. Regarding to negotiate condom use with sexual partners, studies relate many social and cultural barriers. The perception of pleasure, normative beliefs, lack of precise information, gender disadvantage, stereotypical gender roles and communication barriers have been shown to adversely affect lifetime condom use, which contribute to discontinuation of this method [39, 40]. Consequently, this scenario stands as a barrier to the prevention of STIs. On the other hand, discontinuation of hormonal contraception observed was related to side effects, such as also noticed in the literature [35, 41]. A study conducted with hormonal contraceptive users in nine countries reported that switching from oral pills because of side effects was common among Brazilian women. This suggests that women’s dissatisfaction with their method has relevant consequences for contraceptive use [42].

Women’s satisfaction with the contraceptive method is determined according to the characteristics of the methods itself [43]. In this context, studies suggest that reproductive planning actions should prioritize the provision of LARCs, not only because they are associated with high satisfaction, but also because they are highly effective, avoid the need for frequent visits for resupply, they are highly cost effective, and allow a rapid return to fertility after their removal; although they offer no protection against the STIs [17, 44]. Additionally, the provision of LARCs in the Brazilian public health system could modify the Brazilian contraceptive mix, with positive effects in decreasing the occurrence of unintended pregnancies and induced abortions [43].

Despite our selection of a relatively privileged segment of the Brazilian population, we found increased odds of pill disruption among students with lower socioeconomic status. Lower income women are less likely to use contraception compared to women who are better off financially [45]. In Brazil, women have free access to contraception through the public health system, yet delays in appointment may contribute to contraceptive gaps or switches to non-prescription contraception. Although not legally authorized, many women obtain their pills from pharmacists with no prescription, but out of pocket cost may not be an option for the poorest students. In this context, we pointed out that the educational level was not able to void the effect of the socioeconomic status, and this outcome is important in a country as unequal as Brazil. Further investigation of the differential reasons for pill discontinuation by socioeconomic status would shed more light on these findings and inform policy decisions regarding improvement of the quality of family planning services in primary health care.

Another important finding was the association between age and method abandonment. Consistent with our results, study that use data of the National Survey of Family Growth observed that condom users who were younger were almost 42% more likely to discontinue the method compared to older women [28]. Additionally, the authors observed that condom users, who never became pregnant, were 27% less likely to discontinue for method related-reasons when compared to women with children, which is also compatible with our results about the parity and contraceptive discontinuation [28].

Finally, educational background was associated to discontinuation. Pill users who were studying out of the city of São Paulo were 20% less likely to discontinue. This finding was surprising, since São Paulo has more 24 h pharmacies available and primary health care facilities. This is the first study that addressed educational characteristics in the context of discontinuation and more qualitative research is needed to understand the associations observed.

The high frequency of discontinuation observed in our population calls for greater attention to the difficulties Brazilian women face when using short acting methods. Implants and hormonal intrauterine device are only available in Brazil at private clinics and cooper intrauterine device are rarely offered in the public health service [1], leaving women with suboptimal options to prevent a pregnancy. This is particularly concerning in a country that does not legally provide access to abortion when contraception fails.

Contraceptive discontinuation is a particularly significant issue for adolescents and young women, since they tend to have more limited access, and face more barriers than older women in family planning programs. Furthermore, they have more unpredictable and irregular sexual activity; therefore, high quality family planning services are extremely necessary. Adequate, accurate and detailed information on the importance of consistent contraceptive use should be emphasized in order to minimize contraceptive discontinuation, whether during medical consultations or students discussion meetings around the units.

This study has a number of strength and limitations. While the use of probability sampling and a relatively high response rate for an online survey increased the internal validity of the study, our sample is not representative of all Brazilian undergraduate students, such as those at private universities and less educated women. In spite of several incentive programs that favor the inclusion of students from less privileged socioeconomic status at University of São Paulo, studies carried out in some campuses at University of São Paulo observed that the majority of young students is white, belongs to high/medium socioeconomic status, studied in private schools, and the parents of these students have a college degree [46, 47]. Thus, the profile of the young students who participated in this study differs from the other Brazilian youths; however, these are valuable findings and clarify what is happening with this relatively privileged population.

The frequency of discontinuation in this segment of the population calls for population based research to assess the frequency of the issue across the socioeconomic spectrum in Brazil. In this study, we used calendar data to assess monthly use of contraception, allowing for a refined analysis of discontinuation. Retrospective data however, may suffer from recall bias. Additionally, we analyzed only 12-months, nevertheless the recall bias about sexual and contraceptive behaviors may have been minimized by the fact that the study population was composed of young women who initiated the sexual intercourse recently.

## Conclusion

Contraceptive discontinuation is a frequent event among undergraduate students in Brazil, who mostly rely on short-acting methods for pregnancy prevention. Contraceptive discontinuation does differ by type of contraceptive method, condom users were more likely to discontinued their method for method-related reasons, whereas oral pill users were more likely to abandonment or switching to a less effective method. The partnership context was the strongest predictor of discontinuation, emphasizing the importance of relational factors contributing pregnancy risk exposure. Relationship type contributes to method choice, and method discontinuation. The limited contraceptive options in the public health system in Brazil probably reflect the difficulty for women to maintain contraceptive use, and actions that promote the provision of long-term methods should be taken into account in order to minimize the impacts of conceptive discontinuation. We believe that the public health impact of contraception will not be concretized until all women who desire to prevent pregnancies are using their method of choice continuously and effectively.

## Data Availability

The datasets used and/or analyzed during the current study are available from the corresponding author on reasonable request.

## Abbreviations

DHS: Demographic and Health Survey
GEE: Generalized Estimating Equation
QIC: Independence Model Criterion
STIs: Sexually Transmitted Infections
U.S.: United States
